# Factors associated with the prevalence of periodontal disease in low-risk pregnant women

**DOI:** 10.1186/1742-4755-9-3

**Published:** 2012-01-24

**Authors:** Marianna Vogt, Antonio W Sallum, José G Cecatti, Sirlei S Morais

**Affiliations:** 1 Department of Prosthodontics and Periodontics. School of Dentistry of Piracicaba, University of Campinas, Piracicaba, Brazil; 2 Department of Obstetrics and Gynecology. School of Medical Sciences, University of Campinas, Campinas, Brazil

**Keywords:** oral health, periodontal disease, pregnancy, risk factors

## Abstract

**Objective:**

To evaluate the prevalence of periodontal disease (PD) among Brazilian low-risk pregnant women and its association with sociodemographic factors, habits and oral hygiene.

**Method:**

This cross-sectional study included 334 low-risk pregnant women divided in groups with or without PD. Indexes of plaque and gingival bleeding on probing, probing pocket depth, clinical attachment level and gingival recession were evaluated at one periodontal examination below 32 weeks of gestation. Independent variables were: age, race/color, schooling, marital status, parity, gestational age, smoking habit, alcohol and drugs consumption, use of medication, presence of any systemic diseases and BMI (body mass index). Statistical analyses provided prevalence ratios and their respective 95%CI and also a multivariate analysis.

**Results:**

The prevalence of PD was 47% and significantly associated with higher gestational age (PR 1.40; 1.01 - 1.94 for 17-24 weeks and PR 1.52; 1.10 - 2.08 for 25-32 weeks), maternal age 25-29 years, obesity (PR 1.65; 1.02 - 2.68) and the presence of gingival bleeding on probing (OR_adj _2.01, 95%CI 1.41 - 2.88). Poor oral hygiene was associated with PD by the mean values of plaque and bleeding on probing indexes significantly greater in PD group.

**Conclusions:**

The prevalence of PD is high and associated with gingival bleeding on probing, more advanced gestational age and obesity. A program of oral health care should be included in prenatal care for early pregnancy, especially for low-income populations.

## Background

Periodontal disease is a common oral infection with prevalence ranging from 10-60%, and refers to gingivitis and periodontitis [1]. Gingivitis is an inflammatory condition of the soft tissues surrounding the teeth and periodontitis involves localized increases in the numbers and tissue invasion of anaerobic Gram-negative bacteria, causing persistent inflammation and destruction of the supporting structures of the teeth, such as the periodontal ligament and alveolar bone, resulting in mobility and occasional teeth loss [2]. PD involves both direct tissue damage caused by bacterial plaque, accumulated due to a poor oral hygiene, and indirect damage through host inflammatory and immune responses.

Factors including the host's systemic status should be studied since they may affect the prevalence, progression and severity of PD. Sex hormones have been indicated as important modifying factors that may influence the pathogenesis of PD [3].

During pregnancy, progesterone levels increase 10-fold and estrogen levels 30-fold compared to those observed on menstrual cycle due to their continuous production. Physiological changes in metabolism include oral microbial species, immune response and cell metabolism. The increase in progesterone results in greater vascular permeability, gingival edema, crevicular fluid levels and prostaglandin production, which may lead to gingival inflammation [4]. In addition, may affects the development of local inflammation, reducing regulation of interleukin-6 production and rendering gingival tissues less resistant to inflammatory challenges caused by bacteria [5].

Then, in fact there is a relationship between pregnancy and periodontal status [6-11]. Studies have shown that pregnancy does not cause PD but may exacerbate preexisting unfavorable periodontal conditions [9,12]. The depth of periodontal pockets may increase as pregnancy progresses [10,12]; however, the level of activity of the disease does not necessarily result in loss of periodontal clinical attachment level [12]. There is, nonetheless, a consensus that pregnant women suffer a decline in periodontal health status. In spite of some studies showing no association between PD and adverse perinatal outcomes [13,14], a growing number of studies indicate that the consequences of PD activity during pregnancy may affect delivery outcomes, contributing towards prematurity, neonates with low birth weight, small for gestational age and fetal growth restriction [1,15-18].

Based on clinical observations, the prevalence of PD during pregnancy varies from 35% in some studies to 100% in others [4,19]. This variation may reflect the different populations studied and their characteristics, besides the differences in definitions of PD between studies. Speculations have been made on the effects of hormonal changes, systemic health and sociocultural characteristics, as well as other possible factors, on periodontal condition during pregnancy [7,8,10,12,20,21].

Recent scientific publications concentrate on a different aspect of the consequences of the increased PD rates during pregnancy. They focused on preventing exacerbation of periodontal conditions and possible treatment, not only because the disease could interfere with systemic health but also as a way to improve oral health status. There is still limited information about the periodontal conditions of Brazilian pregnant women and more representative epidemiologic studies are necessary. Therefore, the objective of this study was to evaluate the periodontal status of a sample of Brazilian low-income and low-risk pregnant women, assessing the full-mouth prevalence, extent and severity of clinical attachment loss, besides the other clinical parameters, to measure the prevalence of PD and to investigate its possible association with some sociodemographic factors, habits and oral hygiene.

## Materials and methods

### Study design

This is a cross-sectional study that assesses the results of a periodontal evaluation, the prevalence of PD and its association with sociodemographic factors, habits and oral hygiene, performed with low-income and low-risk pregnant women (absence of severe systemic pathological conditions which could characterize high risk pregnancy: diabetes, severe hypertension, other chronic disease) receiving prenatal care at the maternity of the University of Campinas, Brazil, who voluntarily agreed to participate in the study after signing an informed consent form. The study was approved by the Institutional Review Board.

### Study Population

Each one of 334 pregnant women, aged 18 to 42, underwent a single periodontal examination on the day of a scheduled prenatal visit, between February 2004 and August 2005.

### Inclusion of Subjects

Inclusion criteria were: gestational age ≤ 32 weeks and low risk. Women carrying twins, with a greater risk of preterm and/or low birth-weight (cervical incompetence, prior cervical surgery), with a previous preterm and with two or more Caesarean sections were excluded from the study.

Women attending the prenatal outpatient clinic were interviewed by a nurse during the educational support group meetings. The nurse briefly explained the objectives of the study and the procedures involved. Then they were referred directly to the odontological clinic to receive additional information and sign the informed consent form. Immediately afterwards, a questionnaire was filled out for collecting socio-demographic and habits variables, gestational age, and the periodontal examination was done.

### Periodontal examination

It was carried out once during pregnancy before 32 weeks of gestation. The data were recorded on a clinical record form with a complete clinical and periodontal description of all the teeth including third molars. Oral hygiene status was assessed as the percentage of surfaces with plaque, by the dichotomous plaque index (presence or absence of plaque) (PI) [22]. Probing pocket depth (PPD: measurement from the gingival margin to the total probing depth), gingival recession (GR: measurement from the cemento-enamel junction to the gingival margin) and clinical attachment level (CAL: measurement from the cemento-enamel junction to the total probing depth) were evaluated at four tooth surfaces (mesial, buccal, distal and lingual) using a Williams periodontal probe. The greatest clinical measurement of each surface was registered. Bleeding on probing (BOP) was assessed during and recorded after PPD was measured, by the dichotomous index (presence or absence of bleeding), and was expressed as the percentage of surfaces showing bleeding [23]. The examinations were carried out by the same trained periodontist with experience in the field, and an assistant who provided technical support and who filled the data collection forms. The calibration of the exam with another independent professional evaluation or intra-examiner reliability was not performed due to the complaints of the women on the length of time to be spending on that, taking into account the periodontal examination was performed at the same day, just before the prenatal visit. Although this could represent a possible limitation of the study, the procedure was performed with other patients out of the study.

### Criteria of Periodontal Diagnosis

The presence of 4 or more teeth showing at least one site with 4 mm of PPD and clinical attachment loss at the same site, with BOP, was diagnosed as periodontal disease (PD). These criteria were operationally selected for the clinical definition of pregnant women who positively and unequivocally exhibited PD specifically for this study. Therefore two groups of pregnant women were then defined with the purpose to associate them with the socio-demographic and habit variables: one with PD and other without PD. In order to conduct a more accurate evaluation of the characteristics of PD in this population, the extension of the disease was also classified as follows: P1: at least four teeth with PPD and CAL of 4-6 mm; P2: at least four teeth with PPD and CAL of 7-9 mm; and P3: at least four teeth with PPD and CAL of 10 mm. Among the women classified as without PD, those that had BOP in more than 25% of sites were classified as having only gingivitis in some sites, and when it was ≤ 25%, they were classified as having healthy periodontal status [16].

### Data collection

Information was collected from the pregnant women's files and at the time of the exam, by means of a questionnaire, in order to identify the predictor/independent variables that could be associated with PD in this population. These variables were: socio-demographic variables (age, race/color, years of schooling, marital status, parity, body mass index-BMI-estimated with the pre pregnancy weight, and any systemic disease), habit variables (smoking, alcohol and drug consumption, use of medication-any amount regularly), gestational age at examination (weeks), besides some self-reported oral variables.

### Statistical analysis

When each case was finished, with the information on periodontal examination and socio-demographic and habits factors, the form was checked for completeness and correctness. The information was entered to feed a computer database specifically prepared for this study. Consistency tests were then performed to identify errors with are corrected after checking the correspondent information in the clinical records. For the analyses, the frequency distribution of variables was initially evaluated in the two groups and the χ^2 ^test was used to measure statistical significance. Prevalence ratios (PR with their respective 95% CI) were estimated for the different categories of each possible variable evaluated. Student's parametric t-test was used to compare BOP and PI. Next, a multiple logistic regression analysis was carried out that included PD as the outcome and all others as independent variables. The level of significance was established at 5% and Epi.Info 6.1 and SAS were used for the statistical analysis procedures.

## Results

A total of 334 pregnant women were included in the study. Among these, 157 were classified as with PD, and 177 without PD, what represents a 47% prevalence of exposure of PD in this population (Table 1). The ages of women evaluated ranged from 19 until 42 years old. Of all the possible socio-demographic, gestational and habit/behavioral factors evaluated (Table 2), the only showing a statistically significant associations with PD were: more advanced gestational age at the time of the periodontal evaluation, maternal age between 25 and 29 years and obesity (BMI > 29). Being black was at the limit of statistical significance.

**Table 1:** Percent distribution of pregnant women according to the periodontal status based on clinical attachment level (CAL)

Periodontal Category	N	%
Without PD	177	53
With PD	157	47
P1 (4-6 mm)	133	39.8
P2 (7-9 mm)	20	6.0
P3 (≥ 10 mm)	4	1.2
Total	334	100.0

**Table 2:** Distribution of women according to some socio-demographic, habit, pregnancy related and body weight variables and periodontal disease

Variables	Periodontal Disease		PR (95% CI)
		
	With n (%)	Without n (%)	
Age			χ^2 ^= 5.64 p = 0.34
≤ 19	13 (8.3)	28 (15.8)	1
20-24	41 (26.1)	43 (24.3)	1.16 (0.73 - 1.86)
25-29	55 (35.0)	50 (28.2)	1.65 (1.02 - 2.68)
30-34	25 (15.9)	32 (18.1)	1.38 (0.81 - 2.37)
35-39	15 (9.6)	17 (9.6)	1.48(0.83 - 2.64)
≥ 40	8 (5.1)	7 (4.0)	1.68 (0.88 - 3.23)

Ethnic origin			χ^2 ^= 7.20 p = 0.126
White	81 (51.6)	95 (53.7)	1
Black	16 (10.2)	9 (5.1)	1.39 (1.00 - 1.94)
Mulatta	57 (36.3)	73 (41.2)	0.95 (0.74 - 1.23)
Others	3 (1.9)	-	-

Schooling			χ^2 ^= 8.95 p = 0.062
Semiliterate	45 (28.7)	38 (21.5)	1
Primary education	53 (33.7)	57 (32.2)	0.89 (0.43 - 1.45)
Secondary education	56 (35.7)	67 (37.8)	0.84 (0.64 - 1.11)
University education	3 (1.9)	12 (6.8)	0.37 (0.13 - 1.03)
Post-graduate	-	3 (1.7)	-

Marital Status			χ^2 ^= 0.98 p = 0.322
Stable partner	147 (93.6)	169* (95.5)	1
No stable partner	10 (6.4)	7 (4.5)	1.26 (0.84 - 1.91)

Parity			χ^2 ^= 2.44 p = 0.294
0	61 (38.8)	79 (44.6)	1
1	48 (30.6)	57 (32.2)	1.05 (0.79 - 1.39)
≥ 2	48 (30.6)	41 (23.2)	1.24 (0.95 - 1.62)
Total	157 (100.0)	177 (100.0)	

Gestational age at examination (weeks)			χ^2 ^= 7.51 p = 0.023
≤ 16	34 (21.7)	62 (35.0)	1
17-24	57 (36.3)	58 (32.8)	1.40 (1.01 - 1.94)
25-32	66 (42.0)	57 (32.2)	1.52 (1.10 - 2.08)

Smoking			χ^2 ^= 1.79 p = 0.408
No	128 (81.5)	153 (86.5)	1
Yes	19 (12.1)	14 (7.9)	1.26 (0.92 - 1.74)
Not during pregnancy	10 (6.4)	10 (5.6)	1.10 (0.70 - 1.73)

Alcohol consumption			χ^2 ^= 0.0067 p = 0.93
No	146 (93.0)	165 (93.2)	1
Yes	11 (7.0)	12 (6.8)	1.01 (0.65 - 1.58)

Drugs			χ^2 ^= 0.22 p = 0.633
Yes	1 (0.6)	2 (1.1)	0.70 (0.14 - 3.52)
No	156 (99.4)	175 (98.9)	1

Use of medication			χ^2 ^= 2.52 p = 0.112
Yes	49 (31.2)	70 (39.5)	0.82 (0.64 - 1.06)
No	108 (68.8)	107 (60.5)	1

Any disease			χ^2 ^= 0.068 p = 0.793
Yes	31 (19.7)	37 (20.9)	0.96 (0.72 - 1.28)
No	126 (80.3)	140 (79.1)	1

BMI**			χ^2 ^= 5.60 p = 0.132
Low (< 19.8)	23 (14.6)	32 (18.1)	0.96 (0.68 - 1.37)
Normal (19.8-26)	81 (51.6)	105 (59.3)	1
Overweight (26.1-29)	22 (14.0)	19 (10.7)	1.23 (0.89 - 1.71)
Obese (>29)	30 (19.1)	20 (11.3)	1.38 (1.04 - 1.82)

Total	157	177	

* Data missing in one case

** Data missing for two cases

BMI = Body Mass Index

Among all the "self-reported" periodontal variables studied, the reference to gingival bleeding was statistically associated with approximately a two-fold increased risk in prevalence of PD, while not using dental floss was associated with a 1.32-times greater risk (Table 3). The mean values of plaque and BOP indexes recorded during the periodontal examination were significantly greater in the group with PD (Table 4). In the multiple logistic regression analysis, the only variable shown to be independently and statistically associated with the prevalence of PD during pregnancy was gingival BOP (adjusted OR 2.01, 95% CI 1.41 - 2.88; data not shown in table).

**Table 3:** Distribution of patients according to some self-reported oral variables and periodontal disease

Periodontal variables informed by women	Periodontal Disease		PR (95% CI)
		
	With n (%)	Without n (%)	
Gingival bleeding	115 (73.2)	79 (44.6)	1.97 (1.49 - 2.61)
Halitosis	59 (37.6)	53 (29.9)	1.19 (0.95 - 1.50)
Breathes through the mouth	66 (42.0)	86 (48.6)	0.86 (0.69 - 1.09)
Brushes teeth up to twice a day	56 (35.7)	68 (38.4)	0.94 (0.74 - 1.19)
Does not use dental floss	108 (68.8)	101 (57.1)	1.32 (1.02 - 1.70)
Does not use mouthwash*	134 (85.3)	150 (84.7)	1.03 (0.74 - 1.42)
Grinds her teeth	25 (15.9)	40 (22.6)	0.78 (0.56 - 1.09)
Gingival treatment**	22 (14.0)	15 (8.5)	1.31 (0.98 - 1.76)
Eats sweets	40 (25.5)	58 (32.8)	0.82 (0.63 - 1.08)
Total	157 (100.0)	177 (100.0)	

* data missing for one case

** data missing for two cases

**Table 4:** Mean values of periodontal indexes determined by examination, according to periodontal disease

Periodontal Examination	Periodontal Disease			
			
	With	Without	t*	p
PI - Plaque index (%)	73.8 ± 14.2	59.6 ± 17.9	7.99	<0.00001
				
BI - Bleeding on probing index (%)	74.9 ± 14.9	56.7 ± 16.0	10.7	<0.00001
				
Number of teeth	26.8 ± 4.9	27.2 ± 3.3	0.75	0.488
Total	157	177		

* Student's t-test.

There were 157 diagnosed cases with PD, the majority of them (133; 39.8%) with 4-6 mm of attachment loss. Of the 334 women evaluated, all were found to have any gingival BOP at any site, in both groups, with or without PD. Nine women (2.7%) were found to have a BOP index ≤ 25%, while 325 cases (97.3%) had an index > 25%. Among the 177 women classified as without PD in this study (53%), only 16 women (4.8%) had no periodontal attachment loss at all; among these, 9 cases were found to have a BOP index > 25%, gingivitis, while 7 cases (2.1%) had this index ≤ 25%; 161 women (48.2%) had some attachment loss < 4 mm at some site. These results show that only 2.1% of the entire sample of pregnant women in this study had a real oral health status, without any attachment loss at any site or gingivitis (bleeding on probing index ≤ 25%) (data not shown in table).

## Discussion

The current study found a high prevalence of PD during pregnancy of 47% showing a poor periodontal condition of this low-income Brazilian pregnant women sample. Other South American study [16] reported a lower prevalence of PD of 29.85% in pregnant Chilean population. Any gingival bleeding on probing was observed in 100% of this population, confirming findings of some previous studies [6,19,20]. Bleeding on probing index was > 25% in 97.3% of women. This data is in agreement with the findings of Miyazaki *et al. *[19] and Lieff *et al. *[21], who found that in more than 90%. This high prevalence of PD is in accordance with moderate and severe PD forms. The 4 cases of P3 would be classified as aggressive forms of periodontitis [24].

More cases of periodontal damage were detected when the examination was performed later in gestation. These findings are in agreement with data in the literature showing an exacerbation in pre-existing periodontal status as pregnancy progresses [8,11,20]. The study by Machuca *et al. *[7] found greater clinical attachment loss in pregnant women evaluated in the third trimester. Since only one periodontal examination was carried out in the present study, it is not possible to affirm to what extent PD was exacerbated during pregnancy, nor whether there was any clinical attachment loss during this period. It was, however, possible to conclude that periodontal status in this population is bad and that the worst conditions were diagnosed later in pregnancy.

Some studies in which more than one examination was performed during pregnancy showed no clinical attachment loss during the interim period. Tilakaratne *et al. *[8] reported a progressive increase in levels of gingival inflammation throughout pregnancy but no significant attachment loss and concluded that the elevated hormone levels in pregnancy probably affect the gingival more than periodontal attachment. Yalcin *et al. *[20] followed up women during pregnancy and observed constant increases in the indexes of plaque, BOP and PPD, although counseling on oral hygiene was provided throughout the study. Taani *et al. *[10] compared pregnant and non-pregnant women and observed significant increases in bleeding indexes and PPD throughout pregnancy but no significant differences in periodontal clinical attachment loss between the two groups.

Two recent studies found positive results with respect to clinical attachment loss during pregnancy. Although the increase in PPD may represent reversible changes previously documented in pregnant women [21], increases in attachment loss suggest irreversible periodontal damage. Moss *et al. *[12] reported risks for the occurrence and progression of gingivitis/periodontitis during pregnancy and observed that sites with PPD ≥ 4 mm and BOP had a greater probability of suffering an increase in PPD and CAL during pregnancy.

A significant association was found between obesity (BMI > 29) and PD. Although this has not been a focus of other studies on PD in pregnancy, our results are in agreement with those from other studies in non-pregnant populations, whom reported a higher risk of obese subjects having periodontitis [25,26]. BMI is positively correlated with the severity of periodontal attachment loss and this relationship may be modulated by insulin resistance. The fat-distribution, general and abdominal obesity seems to be associated with an increase of PD, besides the maintaining of normal weight by regular physical activity is associated with a lower prevalence of PD. The underlying biological mechanisms for this association are not well known; however, adipose tissue-derived cytokines or adipocytokines, may play a key role in this link by modulation periodontitis [27].

Self-reported gingival bleeding was statistically associated with an approximately two-fold risk of having PD; while the non-use of dental floss was associated with a 1.32-fold risk. These findings are in agreement with the greatest mean values found for the BOP and plaque indexes in the group with PD. Sarlati *et al. *[28] also showed a strong positive correlation between gingival bleeding, PPD and CAL, and an inverse relationship between education level and plaque index in pregnant women. In the current study, besides the high prevalence of PD, the PPD and plaque index levels were high in both groups, demonstrating the precarious state of oral hygiene in this low-income Brazilian pregnant population, just confirming the evidence for oral hygiene (plaque) being a risk factor for PD ^28^. The low-income seems to be an important factor associated with poor oral health in Brazilian studied subpopulations [29], witch present a high prevalence and severity of periodontal breakdown, as well as periodontal inflammation and poor oral hygiene [30].

Young low-income pregnant women of this sample, with age between 25 and 29 years old, showed association with PD, reflecting a social health oral concern, considering its progression if not treated [31]. The hormonal influence of pregnancy on the periodontum may be minimized by good plaque control achieved through an appropriate oral hygiene program. However, the real mechanism through which these interactions actually occur is yet to be fully clarified [3]. Nevertheless, the presence of plaque and preexisting gingival inflammation seems to be prerequisites for the subclinical hormonal changes to be able to initiate progression to severe gingivitis [8].

In the multiple logistic regression analysis, the only independent variable that proved to be statistically associated with the prevalence of PD during pregnancy was the presence of gingival BOP. This finding is in agreement with data reported in the literature that gingival BOP is the most consistent factor found during active progression of the PD and that it may be a predictor of future clinical attachment loss, mainly when associated with greater PPD [32-34].

Even if new periodontal pockets appear and revert after pregnancy [12], it is important to take into consideration that preventing inflammation during pregnancy should be a therapeutic objective. Although treatment and prevention of PD are known to improve oral health status during pregnancy, no studies have yet shown that these therapies are definitively able to reduce adverse delivery outcomes [35,36]. Therefore, it is of clinical interest to evaluate individual risks of changes in PD during pregnancy so that adequate planning of periodontal therapy may be implemented [35].

A program of oral health care should be developed and implemented for early pregnancy, especially for low-income populations. It should provide information on oral hygiene and periodontal treatment during prenatal care, particularly among women at greater risk. If this is true for low risk pregnant women we could probably assume that it would also be the case for high risk pregnancy as well. Although a study published in the literature has reported a reduction in gingival inflammation occurring three months following delivery [8], oral health during pregnancy is important in order to minimize possible undesirable perinatal results and to improve the quality of life and wellbeing of the expectant mother and her baby.

## Abbreviations

BMI: body mass index; BOP: Bleeding on probing; CAL: clinical attachment level; GR: gingival recession; PD: periodontal disease; PI: plaque index; PPD: Probing pocket depth; PR: prevalence ratio.

## Authors' contributions

MV, AWS and JGC participated in all steps of the study, including research planning, data collection, analysis and writing the manuscripts. SSM performed the statistical analysis. All authors gave suggestions, read the manuscript carefully, fully agreed on its content and approved its final version.

## Competing interests

The authors declare that they have no competing interests.
